# Mineral Properties and Dietary Value of Raw and Processed Stinging Nettle (*Urtica dioica* L.)

**DOI:** 10.1155/2013/857120

**Published:** 2013-05-16

**Authors:** Laban K. Rutto, Yixiang Xu, Elizabeth Ramirez, Michael Brandt

**Affiliations:** ^1^Alternative Crops Program, Agriculture Research Station, Virginia State University, Petersburg, VA 23806, USA; ^2^Food Processing and Engineering Program, Agriculture Research Station, Virginia State University, Petersburg, VA 23806, USA; ^3^College of Agriculture and Life Sciences, Virginia Polytechnic Institute and State University, Blacksburg, VA 24061, USA

## Abstract

Stinging nettle (*Urtica dioica* L.) has a long history of usage and is currently receiving attention as a source of fiber and alternative medicine. In many cultures, nettle is also eaten as a leafy vegetable. In this study, we focused on nettle yield (edible portion) and processing effects on nutritive and dietary properties. Actively growing shoots were harvested from field plots and leaves separated from stems. Leaf portions (200 g) were washed and processed by blanching (1 min at 96–98°C) or cooking (7 min at 98-99°C) with or without salt (5 g·L^−1^). Samples were cooled immediately after cooking and kept in frozen storage before analysis. Proximate composition, mineral, amino acid, and vitamin contents were determined, and nutritive value was estimated based on 100 g serving portions in a 2000 calorie diet. Results show that processed nettle can supply 90%–100% of vitamin A (including vitamin A as *β*-carotene) and is a good source of dietary calcium, iron, and protein. We recommend fresh or processed nettle as a high-protein, low-calorie source of essential nutrients, minerals, and vitamins particularly in vegetarian, diabetic, or other specialized diets.

## 1. Introduction

Stinging nettle (*Urtica dioica* L.) has a long history as one among plants foraged from the wild and eaten as a vegetable [1, 2]. Although not fully domesticated, the species remains popular even in the current era for food and medicine as reported, for example, in Nepal [2] and Poland [3].

Despite *U. dioica* being recognized as an edible and highly nutritious vegetable, research attention has focused more on its value as a source of alternative medicine and fiber. Clinical trials have confirmed the effectiveness of nettle root and saw palmetto (*Serenoa repens *(Bart.) Small) fruit extracts in the treatment of benign prostatic hyperplasia [4]. Dried nettle leaf preparations are also known to alleviate symptoms associated with allergic rhinitis [5], and a technology for granulating lipophilic leaf extracts for medicine has been developed [6]. A recent report from ongoing work in Italy confirms the potential of *U. dioica* as a sustainable source of textile fiber [7]. 

There are a number of reports that address the role of *U. dioica* in human nutrition. Fatty acid and carotenoid content in leaf, stem, root, and seed samples have been measured [8], and the properties of phenolic compounds in leaves, stalks, and fibers have been reported [9]. Furthermore, the quality and safety [10] and microbiological properties [11] of sucuk, a Turkish dry-fermented sausage, incorporating dried *U. dioica* leaf have been studied, and the capacity of nettle extracts to improve oxidative stability in brined anchovies has been reported [12]. In the Basque region of Spain, young shoots are reportedly eaten raw or included in omelets [13]. In terms of postharvest processing for long-term storage, microwave drying at 850 W was found to be the best method for preservation of leaf color, energy consumption, and processing time [14]. Mineral content [15] and trace metal concentrations [16] in nettle leaf tea made by infusion or decoction have also been determined. 

However, nettle is consumed primarily as a fresh vegetable whereby it is added to soups, cooked as a pot herb, or used as a vegetable complement in dishes. In this sense, more work needs to be done on nutritive value of fresh nettle, and the fate of minerals and bioactive compounds in processed products. This information is essential because the capacity of fresh nettle to irritate bare skin may discourage potential consumers and postharvest processing methods that make it safe to handle, while maintaining nutritive value will benefit the development of *U. dioica* as a specialty vegetable. 

In this study, we report dietary values, mineral properties, and other quality attributes of raw, blanched, and cooked stinging nettle.

## 2. Materials and Methods

### 2.1. Plant Materials

Plant samples were obtained from field plots planted as a part of an ongoing agronomic study on *U. dioica* at Randolph Farm (37.1°N; 77.3°W), Virginia State University (VSU). Samples from fall and spring growth were collected in October 2011 and May 2012, respectively, by harvesting actively growing shoots (20 ± 2 cm) before the onset of flowering. Individual shoots were clipped with a pair of shears and consolidated in vented plastic bags before transfer to a demonstration kitchen located at the VSU Farm Pavilion for further processing. 

### 2.2. Sample Processing

In the kitchen, the shoots were washed, and twelve 200 ± 5 g units were weighed before separating leaves and tender shoot tips from the woody stem. The edible portion (leaves and tender shoot tips) was weighed, and mean yield was determined by presenting the weight of edible portion as a percentage of total unit mass. Treatments, each replicated three times, were applied as follows: raw samples were packaged and frozen without further processing, blanched samples were immersed in boiling water (98-99°C) for 1 min, and cooked samples were boiled (98–100°C) with or without salt (5 g*·*L^−1^ H_2_O) for 7 min. Both blanched and cooked samples were cooled to 0°C with shaved ice immediately after treatment. All samples were kept in frozen storage (−4°C) before analysis. Samples for proximate composition analysis were submitted frozen, while those for fatty and amino acid analysis were freeze-dried and ground to a fine powder before analysis.

### 2.3. Proximate Analysis

All analysis was done according to the Association of Analytical Chemists (AOAC) methods (AOAC, 2000). Moisture content was determined by drying samples to constant weight using a convection oven. Nitrogen (N) content was measured using a CN analyzer (LECO 528, LECO Corp., St. Joseph, MI), and protein content was derived by multiplying N values with 6.25. Total fat was determined by gas chromatography (Agilent 5890, Agilent Technologies, Santa Clara, CA, USA) after extraction of saponifiable and unsaponifiable fractions, and ash content was measured by ignition at 550°C to constant weight. Carbohydrate content and calorie values were calculated by difference. Total dietary fiber was determined following methods described by the American Association of Cereal Chemists (AACCI method 32-07.01). 

### 2.4. Vitamin and Mineral Analysis

Total vitamin A and vitamin A as *β*-carotene were determined by colorimetry after alkaline digestion followed by extraction with hexane. Vitamin C was extracted in acid and sample content determined by titration. For mineral analysis, samples were subjected to wet digestion before calcium, iron, and sodium content was determined using an ICP spectrometer (AOAC, 2000).

### 2.5. Amino Acid Analysis

For amino acid analysis, a ground subsample of nettle tissue was hydrolyzed with 6 M HCl at 100°C for 24 hr as previously described [17]. Acid hydrolyzed amino acids were derivatized with phenyl isothiocyanate (Acros Organics, Geel, Belgium) and separated using a 2695 Alliance HPLC equipped with a 15-cm Pico-Tag column, 2487 UV/Vis detector, and Empower software (all from Waters Corp., Milford, MA) using previously described conditions [18]. Amino acid concentrations are expressed in g/100 g of nettle leaf. 

### 2.6. Fatty Acid Analysis

Fatty acid methyl esters (FAMEs) were prepared by treating raw and processed samples with ethyl chloride and absolute methanol as described [19]. Fatty acid methyl esters were analyzed by gas chromatography using an Agilent 6890 N GC system (Agilent Technologies), equipped with a HP-INNOWax column (30 m × 0.32 mm I.D. × 0.5 *μ*m film thickness) and flame ionization detector. Peaks were identified against retention times for a known FAME and quantified by the aid of heptadecanoic acid (17:0) included as an internal standard. The concentration of each fatty acid is presented as a percentage of total saponifiable oil in sample.

### 2.7. Statistical Analysis

One-way analysis of variance (ANOVA) using the Analyst function in SAS (version 9.2 for Windows, SAS Institute, Cary, NC) was performed to compare the effects of blanching and cooking on stinging nettle quality and nutritive value. Treatments were treated as independent variables, and data for fall 2011 and spring 2012 were analyzed separately. Tukey's HSD (*P* < 0.05) was used to separate treatment means within season. 

## 3. Results and Discussion

### 3.1. Yield of Edible Portion in *U. dioica*

Actively growing stinging nettle shoots are ideally harvested before flowering for consumption as a potherb or spinach alternative. Leaves on stems were found to be tender enough for use as a vegetable up to 25 cm from the growing point, but stems become woody about 4 cm away from the growing point necessitating destemming after harvest to separate the tender tip (approx. 4 cm and leaves) from the woody stem. Our results show that the woody stem portion accounts for 23%–30% of total biomass with edible portion comprising of 70% or more of harvested material (Table 1). Yield (edible portion) was higher in fall than in spring samples because of seasonal differences in *U. dioica* growth characteristics. Consistent with published observations [20], *U. dioica* displays two distinct phenological stages when grown in south-central Virginia: reproductive growth up to late spring, limited development during summer, and mostly vegetative growth in the fall. 

### 3.2. Effect of Blanching and Boiling on Proximate Composition, Vitamin, and Mineral Content in *U. dioica*

After draining, there was not much difference in moisture content between raw and processed samples in the fall of 2011, while there was slightly more moisture in processed samples in the spring of 2012, likely due to differences in draining time. There was a slight reduction in crude protein, ash, and fat after blanching or cooking in both fall and spring samples. In both cases, the most significant reductions were observed with longer exposure to heat and also to salt. The same applies to dietary fiber, carbohydrate content, and calorie value. Samples harvested in the spring contained significantly higher values for all parameters measured and showed higher decline after processing (Table 2). Preparation and cooking generally result in deterioration of vegetable quality. For example, cooking significantly reduces ash, carbohydrate content, and calorific value in Cocoyam (*Colocasia esculenta*) leaves [21], while chopping amaranth (*Amaranthus* sp.) leaves before cooking can result in increased loss of vitamins and minerals [22]. Our results show that vitamin A, calcium, and iron contents in *U. dioica* leaf are similarly affected by cooking. Sodium content was low and was not affected by cooking, but the salt added to cooking water in one of the treatments significantly (*P* < 0.05) increased sodium content in drained samples (Table 2). Salt addition for seasoning or preservation has been reported to affect vegetable quality through dilution of minerals and other chemical changes [23]. Cooking led to changes in the fatty acid profile of *U. dioica* with more saturated fat being converted into mono-unsaturated and polyunsaturated forms (Table 3) or lost into solution. Saponifiable oil content in raw and processed *U. dioica* samples (3.2%–4.7% in the spring; 3.2%–4.1% in the spring) was comparable to that in wild asparagus (*Asparagus acutifolius*) and black bryony (*Tamus communis*), edible wild greens common to Mediterranean diets [24].

### 3.3. Effect of Cooking on Fatty and Amino Acid Composition in *U. dioica* Tissue Samples

**Figure 1:**
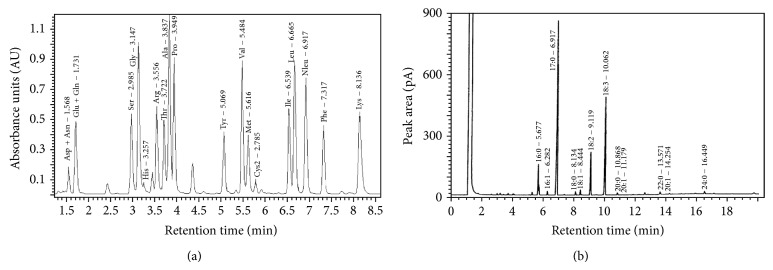
Representative chromatograms showing peaks and retention times for different amino (a) and fatty (b) acids in raw and processed stinging nettle (*Urtica dioica* L.) leaf samples.

Data on individual amino and fatty acid content in stinging nettle shows that the species can supply significant quantities of oleic (18:1), linoleic (18:2), and *α*-linoleic (18:3) acids and is a good source of unsaturated fatty acids. Considerable amounts of palmitic acid (16:0), a saturated fatty acid, were found in the leaf (Table 3; Figure 1). There were no significant differences in fatty acid content between samples collected from fall and spring growth. Similarly blanching and cooking with or without salt did not affect fatty acid content within season except for a general trend showing an increase in unsaturated fatty acid content and a corresponding decrease in the concentration of saturated fatty acids (Table 3). Similarly, high levels of linoleic and *α*-linoleic acids in young and mature leaves and the presence of relatively high concentrations of the same oils in *U. dioica* seed, stem, and roots portions have been reported [8], with the seed containing up to 15% saponifiable oil. 

In terms of omega-3 fatty acid content, *U. dioica* compares favorably with frozen spinach (*Spinacia oleracea* L.) pretreated by steaming, blanching, or autoclaving [25]. Relative to other commonly consumed wild plants, it contains a higher concentration of omega-3 fatty acids than borage (*Borago officinalis*), and about the same level as water-blinks (*Montia fontana*) [26], watercress (*Rorippa nasturtium-aquaticum*), sheep sorrel (*Rumex acetosella*), and sorrel (*Rumex induratus*) [27]. However, carbohydrate content (including total sugars) was significantly lower in raw and processed *U. dioica* (4.2%–16.5%) than in the four species above reported to constitute 66.6%–78.9% total carbohydrates [27]. These results show that processing by blanching and cooking has a minimal impact on *U. dioica* fatty acid composition, implying that it can be a good source of essential fatty acids when eaten as a leafy vegetable. 

With regard to individual amino acids, tissue content was similarly not affected by season. Our results show that *U. dioica* can supply considerable amounts of essential amino acids including threonine, valine, isoleucine, leucine, phenylalanine, and lysine, along with lower concentrations of histidine and methionine (Table 4; Figure 1). Amino acid content was largely unchanged in the spring as compared with fall growth though asparagine, glutamine, leucine, and histidine levels were generally lower in samples from spring growth. There were slight to significant increases in amino acid content after blanching or cooking in fall samples, but no similar observation was made for samples collected in the spring (Table 4). There may be differences between and within species in response to postharvest handling and processing conditions. In one study, a significant increase in amino acid content was recorded after cooking relative to raw spinach [28], while the opposite was true for cooked and frozen versus raw Brussels sprouts [29]. 

Data from this experiment show that both raw and cooked *U. dioica* can be important sources of dietary protein. The species can supply higher concentrations of essential amino acids than Brussels sprouts [29] and has a better amino acid profile than most other leafy vegetables. Although similar to *S. oleracea* in terms of total amino acid content, *U. dioica* contains higher levels of all essential amino acids except leucine and lysine. Some of the published recipes incorporating *U. dioica* leaf flour in bread, pasta, and noodle dough suggest that it can be used as a protein-rich supplement in starchy diets associated with poor and undernourished populations. This is because on a dry weight basis, *U. dioica* leaf is better than almond (dry) and is comparable to common bean (*Phaseolus vulgaris*) and chicken (*Gallus gallus*) as a source of essential amino acids [30]. The agronomic properties of *U. dioica* including perennial growth, quick response to fertilization, and high biomass yield make it an excellent candidate for low-cost mass production for such a purpose.

### 3.4. Labeling Information for Processed *U. dioica*

**Figure 2:**
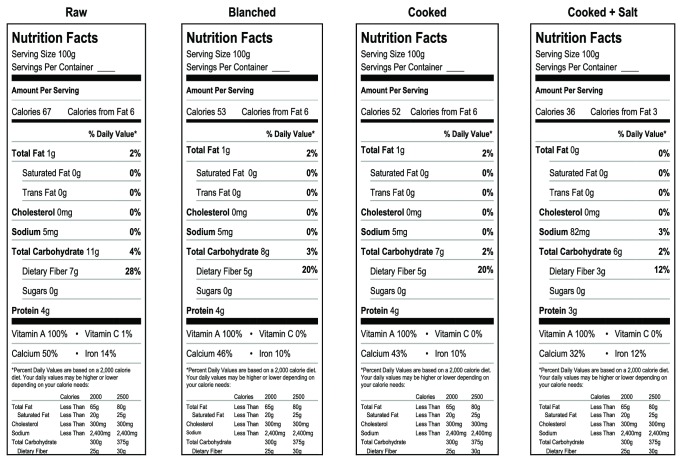
Suggested food labeling information for raw and processed stinging nettle (*Urtica dioica* L.).

 Results from this study show that *U. dioica* retains a significant portion of minerals, vitamins, and essential nutrients after pre-treatment by blanching or cooking prior to frozen storage. Processing may be the most effective approach to availing the nutritional benefits of *U. dioica* to consumers discouraged by the stinging quality of live or fresh nettle. The nutritional information in Figure 2, representing means of data from both spring and fall growth, can be used to label frozen raw and processed *U. dioica* leaf. However, lower vitamin A and higher carbohydrate content and other data reported for blanched *U. dioica* samples collected from the wild [31] show that more work is required to evaluate the properties of *U. dioica* products as affected by interactions between landrace, environment, harvesting time, and processing conditions. 

## 4. Conclusions

Although the usage of *U. dioica* as a leafy vegetable is widespread, there is little information on processing potential, and the impact of different processing methods on nutritive and functional value. The results presented in this report show that *U. dioica* retains significant amounts of minerals, vitamins, and other functional values after blanching or cooking. We recommend processing and selling of *U. dioica* leaf as a highly functional and nutritive food.

## Figures and Tables

**Table 1:** Edible portion (leaf) yield as a percentage of total biomass in stinging nettle (*Urtica dioica* L.) harvested from field plots in the fall of 2011 and spring of 2012. Actively growing shoots (20 ± 2 cm) were harvested and processed by de-stemming.

Season	Shoot wt. (g)	Stem wt. (g)	Leaf wt. (g)	Loss (%)
Fall 2011	203 ± 1.73^a^	46 ± 3.5	157 ± 4.69	23 ± 1.8
Spring 2012	199 ± 5.5	55 ± 7.9	144 ± 10.3	28 ± 4.2

^a^Mean (*n* = 3) ± standard deviation.

**Table 2:** Proximate composition, vitamins, minerals, and fatty acid profile of raw and processed stinging nettle (*Urtica dioica* L.) shoots harvested from field plots in the fall of 2011 and spring of 2012.

	Fall 2011				Spring 2012			
	Raw	Blanched	Cooked	Cooked + salt	Raw	Blanched	Cooked	Cooked + salt
Proximate analysis								
Moisture (%)	89.0 ± 1.4^a^	87.2 ± 0.9^a^	87.7 ± 0.7^a^	88.6 ± 0.5^a^	75.1 ± 1.5^c^	84.6 ± 2.5^b^	85.6 ± 0.8^b^	91.7 ± 0.9^a^
Protein (%)	3.7 ± 0.5^a^	3.6 ± 0.4^ab^	3.6 ± 0.3^a^	2.7 ± 0.2^b^	6.3 ± 0.3^a^	4.1 ± 0.2^b^	3.8 ± 0.3^b^	2.2 ± 0.2^c^
Fat (%)	0.6 ± 0.1^a^	0.4 ± 0.1^b^	0.4 ± 0.0^b^	0.2 ± 0.0^b^	1.4 ± 0.3^a^	1.1 ± 0.1^a^	1.1 ± 0.2^a^	0.6 ± 0.1^b^
Ash (%)	2.1 ± 0.3^a^	1.8 ± 0.3^ab^	1.5 ± 0.3^b^	1.5 ± 0.1^b^	3.4 ± 0.2^a^	1.4 ± 0.1^b^	1.2 ± 0.1^c^	1.0 ± 0.1^c^
Fiber, total dietary (%)	6.4 ± 0.4^a^	4.2 ± 0.1^b^	3.5 ± 0.3^c^	3.6 ± 0.3^bc^	9.7 ± 1.0^a^	5.4 ± 0.9^b^	4.9 ± 1.0^b^	4.2 ± 0.2^c^
Carbohydrates, total (%)	7.1 ± 1.7^a^	6.6 ± 1.4^ab^	6.3 ± 0.8^b^	6.2 ± 1.2^b^	16.5 ± 1.6^a^	8.9 ± 0.7^b^	8.1 ± 1.1^b^	4.2 ± 0.6^c^
Other carbohydrates (%)	2.7 ± 0.2^ab^	2.9 ± 0.3^a^	2.5 ± 0.1^b^	2.7 ± 0.1^a^	6.2 ± 1.0^a^	3.5 ± 0.7^b^	3.3 ± 0.5^b^	2.0 ± 0.1^c^
Calories, total (kcal/100 g)	45.7 ± 3.1^a^	42.6 ± 2.1^a^	44.7 ± 2.5^a^	36.5 ± 2.3^b^	99.7 ± 2.5^a^	62.0 ± 1.0^b^	57.3 ± 1.5^c^	32.0 ± 1.0^d^
Calories from fat (kcal/100 g)	5.0 ± 1.0^a^	4.3 ± 0.6^ab^	2.7 ± 0.5^bc^	2.3 ± 0.6^c^	12.3 ± 1.6^a^	10.0 ± 1.0^ab^	8.7 ± 3.1^b^	4.0 ± 1.0^c^
Vitamins and minerals								
Vitamin A, total (IU/100 g)	4935 ± 104^a^	4851 ± 56^a^	4548 ± 53^b^	4362 ± 78^b^	11403 ± 1333^a^	6470 ± 222^bc^	6021 ± 90^c^	7872 ± 354^b^
Vitamin A, as *β*-carotene (IU/100 g)	5035 ± 213^a^	4689 ± 37^b^	4549 ± 130^b^	4062 ± 39^c^	7860 ± 460^a^	4811 ± 88^b^	5028 ± 65^b^	4154 ± 148^c^
Vitamin C (mg/100 g)	1.1 ± 0.1^a^	0.6 ± 0.1^b^	0.6 ± 0.1^b^	0.5 ± 0.1^b^	0.5 ± 0.0^a^	0.5 ± 0.0^a^	0.5 ± 0.0^a^	0.5 ± 0.0^a^
Calcium (mg/100 g)	278 ± 9^c^	441 ± 12^a^	376 ± 9^ab^	318 ± 52^bc^	788 ± 41^a^	464 ± 10^b^	430 ± 10^b^	316 ± 7^c^
Iron (mg/100 g)	1.2 ± 0.1^c^	1.8 ± 0.2^b^	2.6 ± 0.1^a^	2.5 ± 0.3^a^	3.4 ± 0.3^a^	2.1 ± 0.2^b^	2.1 ± 0.3^b^	1.6 ± 0.1^c^
Sodium (mg/100 g)	5.7 ± 0.1^b^	6.3 ± 0.4^b^	6.5 ± 0.3^b^	87.7 ± 6.0^a^	5.5 ± 0.6^b^	7.0 ± 0.2^b^	6.7 ± 0.2^b^	81.1 ± 2.9^a^
Fatty acid profile								
Saturated fat (%)	35.5 ± 2.6^a^	25.7 ± 2.5^b^	23.6 ± 4.1^ c^	21.7 ± 1.9^d^	32.7 ± 2.8^a^	16.5 ± 1.5^bc^	17.3 ± 1.2^b^	15.7 ± 1.4^c^
Monounsaturated (%)	2.7 ± 0.2^c^	3.3 ± 0.2^a^	4.8 ± 0.3^a^	3.2 ± 0.1^b^	7.5 ± 0.6^a^	5.3 ± 0.3^b^	5.8 ± 1.1^b^	4.6 ± 0.2^c^
Polyunsaturated (%)	61.8 ± 3.5^c^	71.0 ± 2.0^b^	71.6 ± 1.2^c^	75.1 ± 1.9^a^	59.8 ± 2.7^d^	78.2 ± 4.4^b^	76.9 ± 2.5^c^	79.7 ± 2.6^a^
Cholesterol (mg/100 g)	1.0 ± 0.0^a^	1.0 ± 0.0^a^	1.0 ± 0.0^a^	1.0 ± 0.0^a^	1.0 ± 0.0^a^	1.0 ± 0.0^a^	1.0 ± 0.0^a^	1.0 ± 0.0^a^

^a^Mean (*n* = 3) ± standard deviation. Values within a year followed by different letters are significantly different at *P* < 0.05 (Tukey's HSD).

**Table 3:** Fatty acid content^a^ in raw and processed stinging nettle (*Urtica dioica* L.) shoots harvested from field plots in the fall of 2011 and spring of 2012.

	Total fat (%)	Fatty acid^b^ (% of total fat)										
		16:0	16:1	18:0	18:1	18:2	18:3	20:0	20:1	22:0	22:1	24:0
Fall 2011												
Raw	3.15 ± 0.12^c^	17.06 ± 0.05^a^	2.54 ± 0.04^b^	1.86 ± 0.01^a^	2.18 ± 0.01^c^	23.30 ± 0.20^a^	49.55 ± 0.10^d^	0.83 ± 0.01^a^	0.03 ± 0.01^a^	1.37 ± 0.02^a^	0.06 ± 0.01^b^	1.23 ± 0.03^a^
Blanched	4.72 ± 0.05^ab^	14.91 ± 0.12^b^	2.54 ± 0.02^b^	1.41 ± 0.02^c^	2.23 ± 0.02^b^	21.58 ± 0.20^b^	54.42 ± 0.37^c^	0.67 ± 0.01^c^	0.06 ± 0.01^a^	1.11 ± 0.01^c^	0.09 ± 0.02^ab^	0.98 ± 0.01^b^
Cooked	4.65 ± 0.10^b^	14.83 ± 0.09^b^	2.45 ± 0.02^c^	1.60 ± 0.01^b^	1.91 ± 0.03^d^	20.96 ± 0.10^c^	55.48 ± 0.20^b^	0.69 ± 0.01^b^	0.03 ± 0.01^a^	1.13 ± 0.01^b^	0.05 ± 0.01^b^	0.88 ± 0.01^d^
Cooked + salt	4.78 ± 0.14^a^	14.22 ± 0.11^c^	2.62 ± 0.01^a^	1.35 ± 0.01^d^	2.54 ± 0.01^a^	19.67 ± 0.2^d^	56.70 ± 0.34^a^	0.67 ± 0.01^bc^	0.05 ± 0.01^a^	1.13 ± 0.01^b^	0.14 ± 0.01^a^	0.91 ± 0.01^c^
Spring 2012												
Raw	3.17 ± 0.01^d^	16.30 ± 0.04^a^	1.88 ± 0.01^b^	1.76 ± 0.01^a^	2.99 ± 0.02^a^	23.89 ± 0.07^a^	48.06 ± 0.09^d^	1.12 ± 0.01^a^	0.25 ± 0.01^a^	1.63 ± 0.02^a^	0.53 ± 0.01^a^	1.61 ± 0.01^a^
Blanched	4.27 ± 0.04^b^	14.58 ± 0.06^b^	1.70 ± 0.01^d^	1.64 ± 0.01^c^	2.95 ± 0.01^a^	21.56 ± 0.27^b^	53.25 ± 0.30^c^	1.05 ± 0.02^b^	0.20 ± 0.01^c^	1.48 ± 0.01^b^	0.38 ± 0.01^b^	1.26 ± 0.01^d^
Cooked	4.50 ± 0.15^a^	14.07 ± 0.21^d^	1.93 ± 0.03^a^	1.64 ± 0.03^c^	2.30 ± 0.07^c^	20.78 ± 0.35^c^	55.59 ± 0.41^a^	0.99 ± 0.04^c^	0.21 ± 0.01^b^	1.47 ± 0.03^b^	0.38 ± 0.01^b^	1.35 ± 0.12^c^
Cooked + salt	3.58 ± 0.06^ c^	14.29 ± 0.05^ c^	1.84 ± 0.05^c^	1.67 ± 0.03^ b^	2.39 ± 0.01^b^	20.19 ± 0.47^d^	54.44 ± 0.37^b^	1.04 ± 0.02^b^	0.18 ± 0.01^d^	1.50 ± 0.01^b^	0.36 ± 0.01^c^	1.50 ± 0.03^b^

^a^Methylated samples were analyzed for total fatty acid content using gas chromatography.

^b^Palmitic acid (16:0); palmitoleic acid (16:1); stearic acid (18:0); oleic acid (18:1); linoleic acid (18:2); *α*-linoleic acid (18:3); gadoleic acid (20:1); behenic acid (22:0); erucic acid (22:1); lignoceric acid (24:0).

^c^Mean (*n* = 3) ± standard deviation. Column values followed by different letters within season are significantly different at *P* < 0.05 (Tukey's HSD).

**Table 4:** Amino acid content in raw and processed stinging nettle (*Urtica dioica* L.) shoots harvested from field plots in the fall of 2011 and spring of 2012.

Amino acid (g/100 g)	Fall 2011				Spring 2012			
	Raw	Blanched	Cooked	Cooked + salt	Raw	Blanched	Cooked	Cooked + salt
Isoleucine	0.90 ± 0.17^b^	1.13 ± 0.20^ab^	1.30 ± 0.10^a^	1.39 ± 0.06^a^	1.04 ± 0.08^a^	1.04 ± 0.08^a^	1.06 ± 0.09^a^	0.97 ± 0.05^a^
Leucine	1.65 ± 0.27^b^	2.09 ± 0.033^ab^	2.37 ± 0.18^a^	2.56 ± 0.18^a^	1.79 ± 0.38^a^	1.91 ± 0.06^a^	1.91 ± 0.08^a^	1.75 ± 0.03^a^
Lysine	1.11 ± 0.21^a^	1.37 ± 0.11^a^	1.37 ± 0.30^a^	1.48 ± 0.17^a^	1.16 ± 0.38^a^	1.33 ± 0.20^a^	1.19 ± 0.30^a^	1.10 ± 0.19^a^
Methionine	0.24 ± 0.05^a^	0.31 ± 0.04^a^	0.33 ± 0.05^a^	0.35 ± 0.06^a^	0.23 ± 0.15^a^	0.19 ± 0.13^a^	0.17 ± 0.07^a^	0.20 ± 0.13^a^
Tyrosine	0.75 ± 0.13^b^	0.95 ± 0.13^ab^	1.11 ± 0.10^ab^	1.18 ± 0.14^a^	0.97 ± 0.20^a^	0.90 ± 0.10^a^	0.93 ± 0.12^a^	0.91 ± 0.13^a^
Phenylalanine	1.03 ± 0.19^b^	1.27 ± 0.17^ab^	1.43 ± 0.15^a^	1.51 ± 0.03^a^	1.15 ± 0.23^a^	1.14 ± 0.05^a^	1.13 ± 0.04^a^	1.06 ± 0.04^a^
Threonine	1.00 ± 0.17^a^	1.08 ± 0.05^a^	1.12 ± 0.15^a^	1.24 ± 0.08^a^	1.03 ± 0.24^a^	0.75 ± 0.07^a^	0.84 ± 0.11^a^	0.75 ± 0.14^a^
Valine	1.11 ± 0.19^b^	1.40 ± 0.23^ab^	1.60 ± 0.11^a^	1.72 ± 0.16^a^	1.30 ± 0.24^a^	1.28 ± 0.12^a^	1.32 ± 0.15^a^	1.22 ± 0.10^a^
Histidine	0.42 ± 0.09^b^	0.53 ± 0.11^ab^	0.64 ± 0.06^ab^	0.68 ± 0.11^a^	0.32 ± 0.15^a^	0.30 ± 0.12^a^	0.37 ± 0.08^a^	0.22 ± 0.12^a^
Total essential amino acids	8.23 ± 1.36^b^	10.13 ± 1.39^ab^	11.26 ± 1.00^a^	12.11 ± 1.60^a^	8.95 ± 2.14^a^	8.83 ± 0.39^a^	8.93 ± 0.29^a^	8.20 ± 0.59^a^
Arginine	1.22 ± 0.21^b^	1.57 ± 0.27^ab^	1.79 ± 0.16^a^	1.97 ± 0.14^a^	1.55 ± 0.42^a^	1.43 ± 0.26^a^	1.56 ± 0.21^a^	1.52 ± 0.24^a^
Aspartic acid + asparagine	0.85 ± 0.32^a^	1.01 ± 0.25^a^	0.88 ± 0.40^a^	1.01 ± 0.04^a^	0.60 ± 0.37^a^	0.47 ± 0.09^a^	0.49 ± 0.10^a^	0.39 ± 0.14^a^
Glutamic acid + glutamine	1.69 ± 0.39^a^	2.13 ± 0.19^a^	1.97 ± 0.62^a^	2.22 ± 0.26^a^	1.49 ± 0.72^a^	1.25 ± 0.26^a^	1.42 ± 0.13^a^	1.14 ± 0.27^a^
Serine	0.85 ± 0.14^b^	1.06 ± 0.15^ab^	1.14 ± 0.13^ab^	1.26 ± 0.10^a^	1.00 ± 0.29^a^	0.82 ± 0.12^a^	0.96 ± 0.15^a^	0.82 ± 0.20^a^
Proline	0.90 ± 0.15^b^	1.11 ± 0.17^ab^	1.31 ± 0.13^ab^	1.41 ± 0.20^a^	1.24 ± 0.17^a^	1.06 ± 0.22^a^	1.19 ± 0.28^a^	1.07 ± 0.16^a^
Glycine	0.92 ± 0.15^b^	1.13 ± 0.16^ab^	1.26 ± 0.10^ab^	1.39 ± 0.17^a^	1.14 ± 0.23^a^	0.98 ± 0.22^a^	1.12 ± 0.22^a^	0.97 ± 0.12^a^
Alanine	1.20 ± 0.19^b^	1.40 ± 0.13^ab^	1.54 ± 0.11^ab^	1.66 ± 0.16^a^	1.54 ± 0.29^a^	1.24 ± 0.22^a^	1.38 ± 0.26^a^	1.21 ± 0.14^a^
Total amino acids	17.46 ± 2.88^b^	21.58 ± 3.40^ab^	22.87 ± 2.21^ab^	24.76 ± 0.96^a^	19.40 ± 5.00^a^	17.77 ± 1.83^a^	18.73 ± 1.44^a^	16.97 ± 1.63^a^
Dry matter (g/100 g edible portion)	11.0	12.8	12.3	11.4	14.9	15.4	14.4	8.3

^a^Mean (*n* = 3) ± standard deviation. Row values followed by different letters within season are significantly different at *P* < 0.05 (Tukey's HSD).
